# Analysis of cystic hygroma diagnosed in the first trimester: Single-center experience

**DOI:** 10.4274/jtgga.galenos.2019.2019.0032

**Published:** 2020-06-08

**Authors:** Betül Yakıştıran, Orhan Altınboğa, Emre Canpolat, Esra Şükran Çakar, Şevki Çelen, Ali Turhan Çağlar, Yaprak Engin Üstün

**Affiliations:** 1 Department of Obstetrics and Gynecology, University of Health Sciences, Ankara Zekai Tahir Burak Women’s Health Training and Research Hospital, Ankara, Turkey; 2 Department of Pediatrics, University of Health Sciences, Ankara Zekai Tahir Burak Women’s Health Training and Research Hospital, Ankara, Turkey; 3 Department of Genetic, University of Health Sciences, Ankara Zekai Tahir Burak Women’s Health Training and Research Hospital, Ankara, Turkey; 4 Department of Obstetrics and Gynecology, University of Health Sciences Turkey, Ankara Etlik Zubeyde Hanım Women`s Health and Research Center, Ankara, Turkey

**Keywords:** Cystic hygroma, perinatal outcomes, prenatal diagnosis

## Abstract

**Objective:**

To evaluate the obstetric outcomes of fetuses with cystic hygroma other than karyotype abnormalities and structural malformations.

**Material and Methods:**

We conducted a retrospective study based on the review of medical records of pregnant women in whom ultrasonographic diagnosis of fetal cystic hygroma was established in the first trimester from January 2014 to October 2018. All patients were offered genetic counselling and prenatal invasive diagnostic procedures to obtain fetal karyotype. For ongoing pregnancies fetal echocardiography and detailed second trimester sonographic anomaly screening was performed by a perinatologist/pediatric cardiologist. The demographic characteristics of the women and the results of the karyotype analysis were obtained from the database of our hospital and correlated with the obstetric outcomes.

**Results:**

Within a five-year period, there were 106 cases of fetal cystic hygroma. Of those, fetal cardiac malformations were detected in four and micrognathia in one fetus. Eighty-five women underwent fetal invasive procedures and karyotype abnormalities were detected in 52 of the cases. Fetal outcomes of 33 cases with normal karyotype and 21 cases in whom karyotyping analysis were not performed due to patient refusal were enrolled into the study. Obstetric outcomes of 21 women who refused karyotyping consisted of 13 livebirths, seven missed abortions, and one fetal death, whereas those of 33 women with normal karyotype were; 12 livebirths, 12 missed abortions, two hydrops fetalis, and five fetal deaths. Nineteen of 33 fetuses with a normal karyotype and eight of 21 fetuses in whom karyotyping was not performed were terminated.

**Conclusion:**

The presence of cystic hygroma carries a high risk for fetal karyotype abnormalities and cardiac malformations. The postnatal outcomes of the fetuses with cystic hygroma appeared to be correlated with the absence of structural malformations and karyotype abnormalities.

## Introduction

Cystic hygroma is a congenital malformation characterized by the presence of abnormal fluid collection at sites of lymphatic-venous collection within the neck, mediastinum, abdomen, and axillary region (1). It is also defined as a subgroup of lymphangiomas with the cystic variety, filled with protein-rich fluid (2). Cystic hygroma is classified as septated and non-septated. The overall incidence of cystic hygroma is approximately 1/1000-6000 births and 1/750 spontaneous abortions (3).

Cystic hygromas of nuchal origin are reported to be associated with fetal aneuploidy and structural anomalies in 50-80% of cases (1). Association with aneuploidy and/or fetal structural abnormalities worsens the prognosis. This condition can be diagnosed with sonography during fetal nuchal translucency measurement in the mid-sagittal plane in the first or early second trimester. When a cystic hygroma is diagnosed, the fetus should undergo thorough anatomic scanning for other system anomalies. In the next step, the parents should be offered invasive procedures for fetal karyotype analyses in order to detect any chromosomal abnormalities (4). If they accept karyotype analyses, chorion villus sampling or amniocentesis should be performed by a perinatologist.

Previous studies reported poorer perinatal outcomes in pregnancies with fetal cystic hygroma and associated aneuploidy (5,6). We conducted this retrospective study to evaluate the pregnancy outcomes of fetuses with cystic hygroma either with normal karyotype or with no karyotype analysis in the prenatal period.

## Material and Methods

All procedures involved performed in studies involving animals and humans were in accordance with the ethical standards of the institution or practice at which the studies were conducted.

A retrospective cohort based on a review of medical records of patients with fetal cystic hygroma, diagnosed and/or referred to our hospital, between January 2014 and October 2018 was conducted. All scans were performed using a Voluson™ 730 Pro (GE Healthcare, USA) multifrequency convex transducer at 2.0-7.0 MHz. Cystic hygroma was defined as an enlarged sonolucency with clearly visible septations extending along the fetal body axis, in contrast to nuchal translucency, which was described as a non-septated sonolucent area confined to the fetal neck. They were differentiated from nuchal edema by the presence of the nuchal ligament. Upon diagnosis of cystic hygroma, all patients were provided genetic counselling. Prenatal invasive diagnostic procedures were offered for fetal karyotyping. A complete fetal anomaly scanning was then performed for the detection of other associated structural anomalies. Women who wanted to continue their pregnancies with cystic hygroma with normal karyotype and undetermined karyotype/due to the fact that parents did not accept the invasive procedures were enrolled into the study. For those women, fetal echocardiography and second trimester detailed sonographic evaluation were performed by a perinatologist.

The demographic characteristics of the patients, and results of the fetal karyotypes were recorded from the electronic database of the hospital. Pregnancy outcomes were tabulated from electronic records of the hospitals, and for women who did not deliver at our hospitals, telephone interviews for the pregnancy outcomes were made. Moreover, physical examination findings of the infants were also performed by inviting them to the hospital.

### Statistical analysis

Data were calculated using the SPSS 11.5 software package for Windows (SPSS Inc., USA). Descriptive statistics are presented as mean ± standard deviation and median (minimum-maximum) and percentages.

## Results

Within this five-year period, there were 106 cases of fetal cystic hygroma; 85 women underwent karyotype analysis, whereas 21 refused karyotype analysis.

The demographic characteristics of the women are depicted in Table 1. The median maternal age was 35 (range, 22-40) years. Among them, a normal karyotype was revealed in 33 (38.8%) cases. In the remaining 52 (61.2%), fetal karyotype abnormalities were detected and they were excluded from the study. Thus the study population included the outcomes of 54 fetuses with cystic hygroma in whom invasive diagnostic procedures were either not performed (n=21) or normal (n=33).

**Figure 1:**
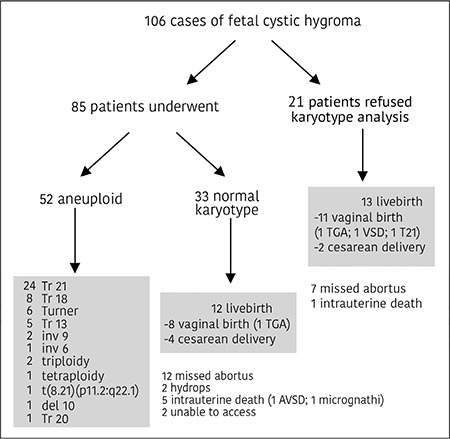
Overall outcomes from the prenatally diagnosed cases of cystic hygroma

The flow chart of the evaluation of the women with hydrops fetalis together with the details of the karyotype abnormalities, fetal structural abnormalities, and pregnancy outcome are summarized in Figure 1. Associated structural anomalies were present in 7 (12.9%) cases, including hydrops fetalis (n=2; 28.6%), transposition of the great arteries (TGA) (n=2; 28.6%), perimembranous ventricular septal defect (VSD) (n=1; 14.3%), atrioventricular septal defect (AVSD) (n=1; 14.3%), and micrognathia (n=1; 14.3%).

In the group that refused karyotype analysis (n=21), pregnancy outcomes were as follows: 13 live births (n=11 vaginal births; n=2 cesarean deliveries), seven missed abortions, and one intrauterine death. In the other group with normal fetal karyotype (n=33), pregnancy outcomes included 12 live births (n=8 vaginal births; n=4 cesarean deliveries), 12 missed abortions, two hydrops, and five fetal deaths. We were unable to obtain the results about pregnancy outcomes in two fetuses with cystic hygroma with normal karyotype. Second trimester pregnancy termination was performed on 19 women with fetal cystic hygroma with normal karyotype and eight of 21 women who refused karyotyping. Two newborns with cardiac malformation died within the first week of delivery. Both of these cases were TGA. Follow-up of the 23 infants continued for nearly 36 months. Only one infant underwent surgery due to congenital hip dislocation. Regarding the 13 live births that refused karyotyping in utero, and were karyotyped postnatally; one infant was trisomy 21 and the remaining 12 infants were euploid. No neurologic developmental disorders were detected in any infants excluding the infant with trisomy 21.

## Discussion

This study once again confirms the fact that increased nuchal translucency in first trimester screening is associated with chromosomal abnormalities, structural malformations, and fetal demise. The overall probability of live births for both groups was 23.5%, and after the exclusion of aneuploid fetuses, it was 46.3%. Bilardo et al. (7) reported these rates as 43.2% and 68.1%, respectively, for increased nuchal translucency groups. On the other hand, the live birth rates of women who refused karyotyping prenatally was 61.9% (n=13/21), and the overall chance of live birth in the total group was 12.3% (n=13/106).

Nuchal translucency is an essential part of the screening for chromosomal anomalies on routine or indicated first trimester fetal sonographic assessment. During fetal nuchal translucency measurement in the mid-sagittal plane, we should keep in mind the association between increased nuchal translucency and chromosomal abnormalities, congenital malformations or several genetic syndromes (8). When a cystic hygroma is diagnosed, detailed ultrasound examination and fetal chromosomal analyses are indicated due to the high rates of fetal aneuploidy and coexisting structural malformations (9). Despite invasive prenatal diagnostic procedures for fetal karyotyping and parental counseling about poor fetal prognosis, parents sometimes refuse these procedures due to religious beliefs, and the increased risk of abortion with invasive fetal procedures. Even if normal karyotype is reported due to the limited treatment modalities and possibility of unfavorable consequences, the parents can opt for elective termination.

Previous studies indicated that cystic hygroma with fetal structural abnormalities is associated with poor fetal outcomes (1,2,3,4,5,6). The most frequent fetal structural malformation is cardiac defects within euploid groups. TGA has the highest incidence (90%) in this group. Septal or valvular defects are present in 43% and aortic valve/isthmus stenosis is present in 86% of cardiac abnormalities (7). We found that the overall frequency of cardiovascular anomalies in our study group was 7.4%. Among the cardiac defects, TGA, perimembranous VSD, and AVSD were detected with a frequency of 28.6%, 14.3%, and 14.3%, respectively, emphasizing the fact that in the second trimester, targeted fetal echocardiographic examinations are important and an essential diagnostic tool in euploid fetuses with cystic hygroma (1,7).

Hydrops fetalis is another important prognostic marker. Bernard et al. (10), reported a mortality rate of 96.5% in hydropic fetuses. Our findings showed that the incidence of fetal death with coexisting hydrops was 100%. Generalized edema and hydrops may be the cause of left atrium dysfunction and aorta due to a compression effect leading to fetal death. In the literature, only a few studies have reported the resolution of hydrops and healthy newborns (11); the majority of the studies demonstrate that hydrops is associated with poor fetal outcomes (6,10,12,13). On the other hand, the resolution of nuchal edema with a normal karyotype is a good prognostic marker in the absence of any coexisting malformation. Two fetuses with hydrops fetalis were present in our study. Cardiac malformations were detected more frequently than hydrops fetalis. Cardiac malformations, arrhythmia, aneuploidy, and fetal structural malformations may lead to non-immune hydrops fetalis (14). Our findings showed 25 live births in all groups, two of which with TGA died postnatally; one of the newborns was trisomy 21. Of the remaining newborns, 22 (88%) had normal postnatal neurologic development. Similarly, Sanhal et al. (5) reported that 90% of fetuses (euploid and structurally normal) with septated cystic hygroma had normal neurologic outcomes.

Other than fetal karyotyping, chromosomal microarray analysis (CMA) is an advanced technology with the ability to survey the entire genome and to identify chromosomal abnormalities, submicroscopic genomic alterations. Increased nuchal translucency and cystic hygroma are associated with different conditions, aneuploidy and structural abnormalities. Shaffer et al. (15) reported that the detection rate of CMA for fetuses with cystic hygroma was 17.1%. CMA should be offered for any patient undergoing invasive sampling to identify all clinically significant alterations.

Though previous studies focused on cases with karyotyping, our study also investigated cystic hygroma with unknown karyotype in the prenatal period. The refusal rate of karyotyping was higher (19.8%) than in the published data from European countries; this condition might be due to lower sociocultural levels and religious beliefs. We think our findings may be helpful to physicians providing parental counseling for women who refuse karyotype analysis. In fetuses with cystic hygroma with normal karyotype and in whom no structural malformations are present, pregnancy outcomes may be favorable as reported in the literature (5).

The small number of cases with cystic hygroma and unknown karyotype in 21 cases are the main limitations of this study.

## Conclusion

The presence of cystic hygroma carries a high risk for aneuploidy and major structural malformations. Invasive prenatal karyotyping procedures, fetal echocardiographic examination, and parental counselling are necessary for the prediction of the prognosis. Until multicenter and large-sample sized studies have been published, these results might be helpful in providing parental counselling for those with fetal cystic hygroma.

## Figures and Tables

**Table 1:**
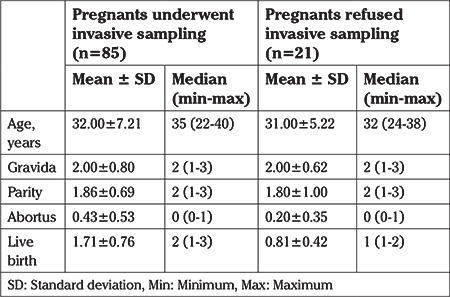
Demographic characteristics of study group
